# Photoacoustic Calorimetry Studies of O_2_-Sensing FixL and (R200, I209) Variants from *Sinorhizobium meliloti* Reveal Conformational Changes Coupled to Ligand Photodissociation from the Heme-PAS Domain

**DOI:** 10.1021/acs.biochem.3c00438

**Published:** 2023-12-21

**Authors:** Audrey Mokdad, EuTchen Ang, Michael Desciak, Christine Ott, Avery Vilbert, Olivia Beddow, Artiom Butuc, Randy W. Larsen, Mark F. Reynolds

**Affiliations:** † Department of Chemistry, University of South Florida, 4202 East Fowler Avenue SCA 400, Tampa, Florida 33620, United States; ‡ Department of Chemistry and Biochemistry, Saint Joseph’s University, 5600 City Avenue, Philadelphia, Pennsylvania 19131, United States

## Abstract

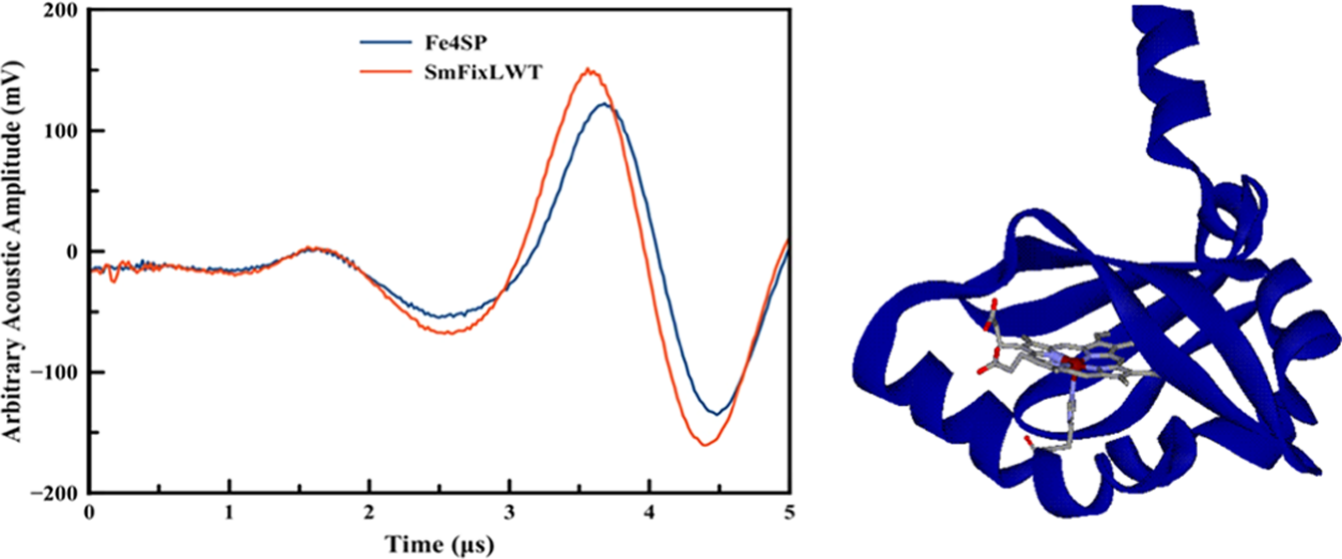

FixL is an oxygen-sensing heme-PAS protein that regulates nitrogen fixation in the root nodules of plants. In this paper, we present the first photothermal studies of the full-length wild-type FixL protein from *Sinorhizobium meliloti* and the first thermodynamic profile of a full-length heme-PAS protein. Photoacoustic calorimetry studies reveal a quadriphasic relaxation for *Sm*FixL*WT and the five variant proteins (*Sm*FixL*R200H, *Sm*FixL*R200Q, *Sm*FixL*R200E, *Sm*FixL*R200A, and *Sm*FixL*I209M) with four intermediates from <20 ns to ∼1.5 μs associated with the photodissociation of CO from the heme. The altered thermodynamic profiles of the full-length *Sm*FixL* variant proteins confirm that the conserved heme domain residues R200 and I209 are important for signal transduction. In contrast, the truncated heme domain, *Sm*FixLH_128–264_, shows only a single, fast monophasic relaxation at <50 ns associated with the fast disruption of a salt bridge and release of CO to the solvent, suggesting that the full-length protein is necessary to observe the conformational changes that propagate the signal from the heme domain to the kinase domain.

## Introduction

A large number of heme proteins have now been discovered in nature that sense O_2_, NO, or CO.^1−8^ These proteins, called heme-based gas-sensing proteins, are found in all kingdoms of life and regulate many important biological processes, such as blood pressure, circadian rhythm, nitrogen fixation, chemotaxis, and photosynthesis.^1−8^ Heme-based gas-sensing proteins are typically classified based on the structural fold of the gas-sensing heme domain and include the heme-PAS (Per-Arnt-Sim) family, the NO sensors (H-NOX), CO-sensing heme proteins (CooA), globin-coupled sensors (GCSs), and the GAF family.^1−8^ In addition, a variety of heme-responsive proteins have been discovered that can reversibly bind heme, and some that are also affected by gases have been identified.^9−12^

**Figure 1:**
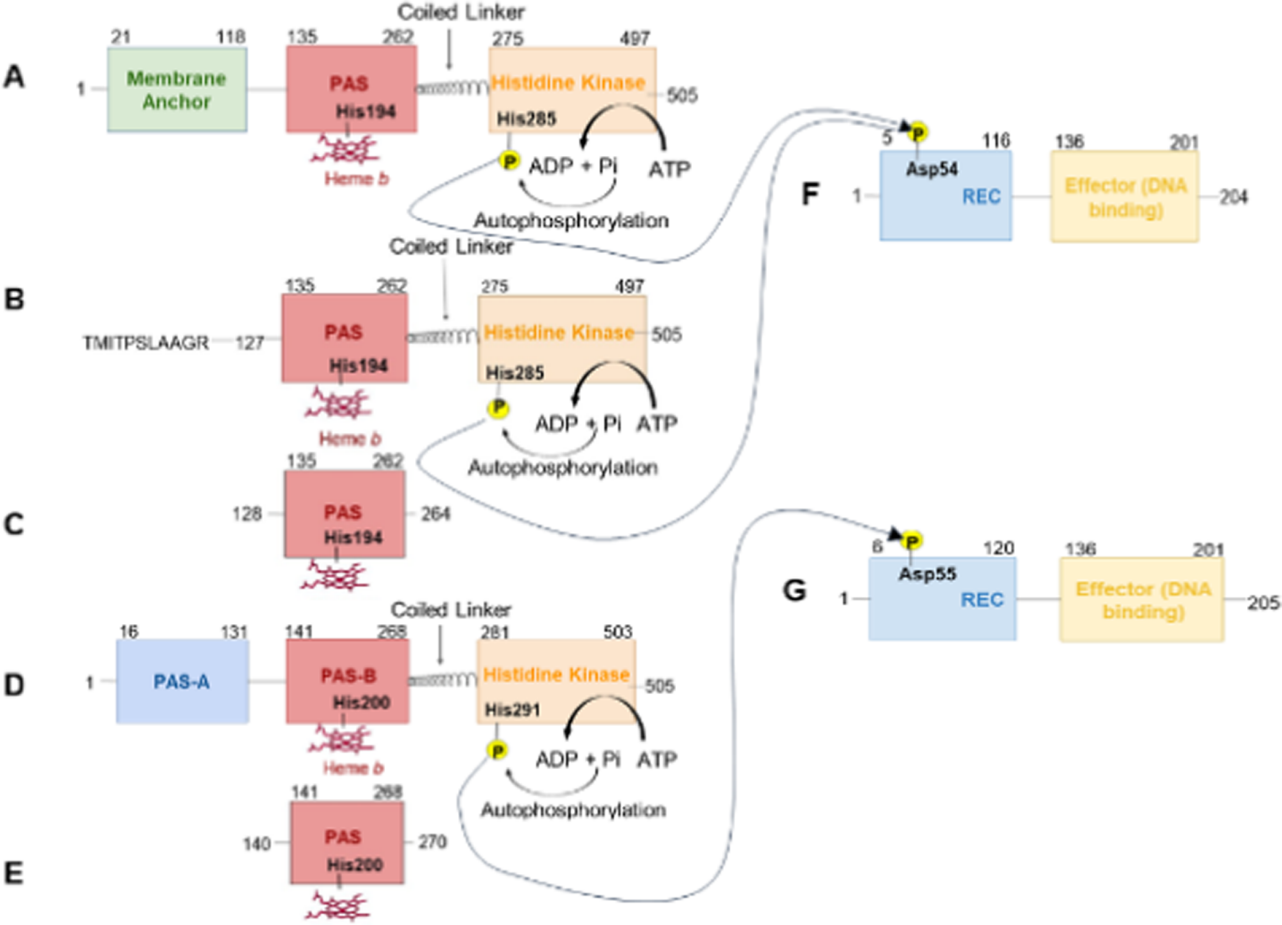
Schematic representation of the domain structures of full-length and truncated versions of the FixL protein. (A) Full-length FixL from *S. meliloti* containing the N-terminal membrane anchor, the N-terminal heme-PAS domain, the coiled linker, and the histidine kinase domains. (B) Full-length *Sm*FixL*_127–505_ which contains the heme-PAS domain, the coiled-coil linker, and the kinase domain but is missing the N-terminal membrane anchor for solubility and instead has an N-terminal extension: (N-TMITPSLAAGR(127–505)-C).^20^ (C) The truncated heme domain of *Sm*FixLH_128–264_. (D) Full-length *Bj*FixL from *B. japonicum* containing the PAS-A and heme-PAS-B domains, the coiled-coil linker, and the histidine kinase domain. (E) The truncated heme domain *Bj*FixLH_140–270_. (F) The response regulator FixJ from *S. meliloti* is phosphorylated by deoxy *Sm*FixLWT and deoxy *Sm*FixL*. (G) FixJ from *B. japonicum* is phosphorylated by deoxy *Bj*FixLWT.

*Sinorhizobium meliloti* (formerly,*Rhizobium meliloti*) and *Bradyrhizobium japonicum* contain a two-component oxygen-sensing system that regulates nitrogen fixation in the symbiotic root nodules of alfalfa and soybean plants, respectively.^13−17^ Two-component sensors are ubiquitous in nature and contain a sensor histidine kinase (HK) domain that is regulated by environmental stimuli and a response regulator (RR) that can stimulate gene transcription.^18−20^ The FixL protein was the first oxygen-sensing heme protein discovered in nature and has been an important paradigm for the structure and function of the heme-PAS family of gas-sensing heme proteins.^1−3,21−24^ FixL from *S. meliloti* contains an N-terminal membrane anchor, an N-terminal heme-PAS domain, a coiled-coil (CC) linker domain, and a C-terminal histidine kinase domain (Figure 1) and forms a dimer in solution.^21−24^

**Figure 2:**
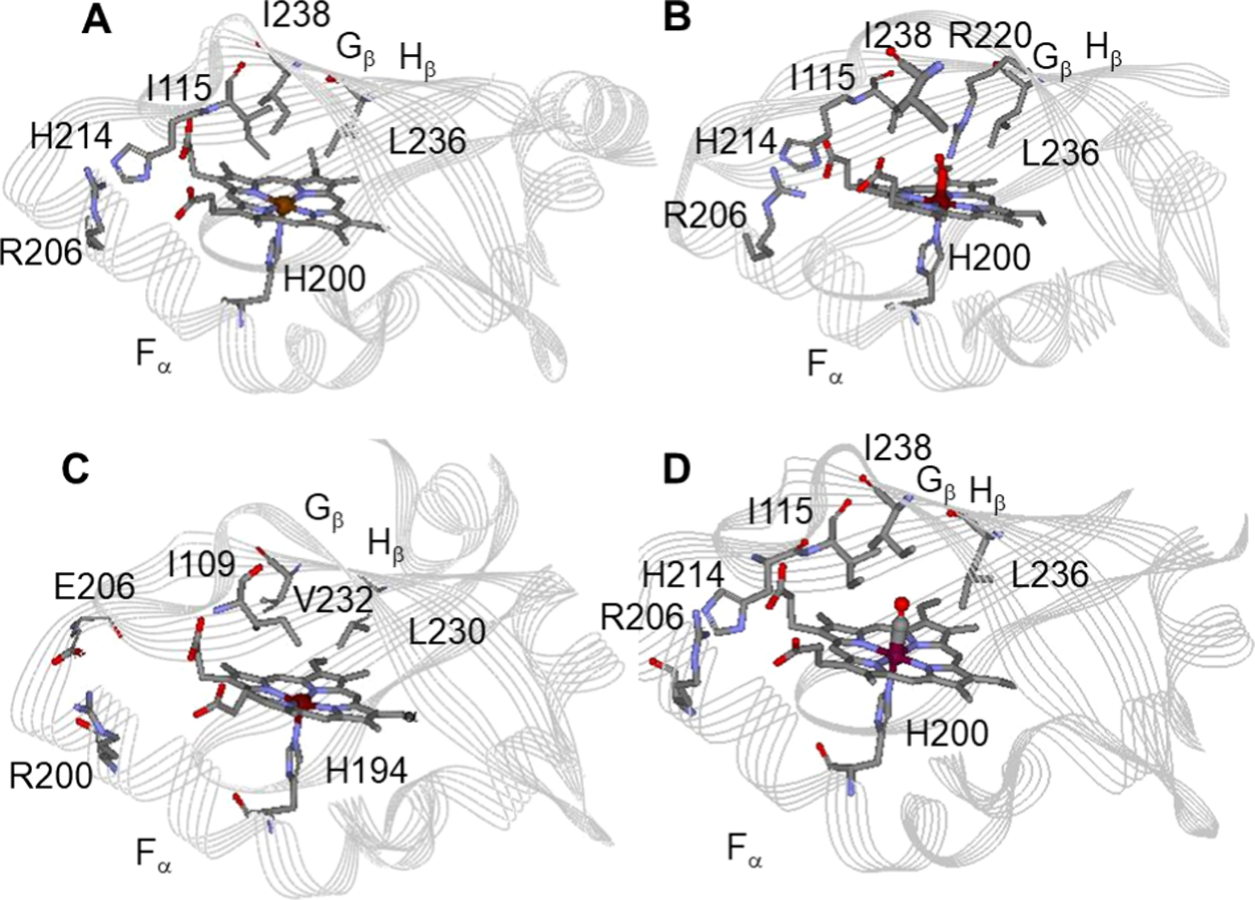
Crystal structures of the truncated heme domains of (A) deoxy *Bj*FixLH_141–270_ (1XJ3), (B) oxy *Bj*FixLH_141–270_ (1DP6), (C) deoxy *Sm*FixLH_127–260_ (1EW0), and (D) the CO-bound structure of *Bj*FixLH_141–270_ (1XJ4).

FixL from *B. japonicum* has a second N-terminal PAS domain instead of an N-terminal membrane anchor. The kinase domain of *Sm*FixL contains a histidine285 (His291 in *Bj*FixL) that is autophosphorylated by ATP (Figure 1). Under hypoxic conditions in the root nodules, O_2_ is released from the heme of dimeric *Sm*FixL, activating the histidine kinase domain, which transfers a phosphate group from histidine285 to an aspartate54 residue on the dimeric response regulator *Sm*FixJ. Phosphorylated *Sm*FixJ triggers the expression of FixK2, which is the transcriptional activator of the nitrogen fixation genes.^13−17^ Under increased oxygen tension, O_2_ binds to deoxy *Sm*FixL, which blocks the phosphorylation of FixJ and expression of the nitrogen fixation genes.^13−17^ The O_2_ analogs NO and CO can also bind to the heme of reduced, deoxy*Sm*FixL and inhibit the kinase domain, but to a lesser extent than O_2_.^25^ Structural and biochemical studies of the ligand-bound and ligand-free forms of FixL and FixJ have begun to reveal the molecular details of this oxygen-sensing system.^26−37^ The truncated heme-PAS domain of deoxy *Sm*FixLH_127–260_ contains a catcher’s mitt structure with the heme sandwiched between the F_α_-helix and several antiparallel β-sheets (Figure 2).^26−37^

The heme domains of *Sm*FixL and *Bj*FixL are ∼53% identical in an amino acid sequence based on UniProt, while the residues in the heme pocket are ∼70% similar. The conserved proximal histidine ligand, His194 in *Sm*FixL (His200 in *Bj*FixL), that binds the heme iron is part of the F_α_-helix, while the hydrophobic β-sheet residues Ile209, Ile210, Leu230, and Val232 cover the distal face of the heme where oxygen binds.^26−37^ A similar heme domain structure is observed for deoxy *Bj*FixLH_141–271_ although there are differences in the hydrogen-bonding network.^26−37^ Ligand binding to the heme of deoxy *Bj*FixLH_141–271_ results in the displacement of the hydrophobic distal residues and the formation of a hydrogen bond between the conserved Arg220 (Arg214 in *Sm*FixL) and the heme-bound ligand, as well as additional changes to the hydrogen-bonding network.^26−37^ The *Bj*FixLH_140–270_ and *Sm*FixLH_127–260_ heme domains are dimeric in solution, analogous to the full-length FixL proteins.^38,39^

Several signal transduction mechanisms have been proposed for FixL based on the available crystal structures and biochemical data for the liganded and unliganded forms.^25−36,40−44^ Gong et al. have observed that in *Bj*FixLH_141–271_, there is a rearrangement of the hydrogen-bonding network between the heme 6,7 propionates and the conserved Arg206 (Arg200 in *Sm*FixL) and His 214 amino acid residues due to the flattening of the heme after binding of the ligand.^27,28^ The *Bj*FixLArg206Ala variant resulted in a significant reduction of the kinase inhibition by O_2_, suggesting that this conserved arginine residue is important for signal transduction.^27,28^ Balland et al. showed that the conserved Arg220 from *Bj*FixL (Arg214 in SmFixL) is important in the oxygen binding mechanism, as it interacts with O_2_ bound to the heme iron.^40^ The displacement of Arg220 or the coordination of a strong field ligand to the heme iron, however, is not responsible for the conformational changes but only the formation of a strong hydrogen-bonding network between Arg220 and the ligand O_2_.^40^ On the other hand, Miyatake et al. have observed that the hydrophobic residues in the distal heme pocket, which likely interact with O_2_, play an important role in signal transduction between the heme and kinase domains.^26^ Thermodynamic profiles for CO photodissociation from the truncated heme domain of *Bj*FixLH_140–270_ by Miksovska et al. demonstrated a biphasic relaxation.^41^ The first phase involved a contraction of the solvent associated with a change in charge distribution after the reorganization of the salt bridge between Glu182 and Arg227 or a possible reorientation of Arg206 after photodissociation of CO from the heme. The second phase with a lifetime of 150 ns was attributed to an expansion due to the ligand being released to the solvent.^41^ Ayers and Moffat, and Cusanovich and Meyer, suggested that the signal originated from the heme domain can be propagated through the linker to the kinase domain by quaternary structural changes via a distortion of the β-sheet based on time-resolved crystallographic studies of the BjFixLH heme domain upon CO photolysis.^42,43^ Finally, Reynolds et al. demonstrated the importance of the conserved, proximal Arg200 in the stabilization of the kinase inhibition related to the oxy form of SmFixL.^44^ They showed a clear relation between the H-bond of Arg200 and the 6-propionate heme group that stabilized the inactive form of *Sm*FixL upon O_2_ binding to the heme.

Although FixL and other heme-based gas-sensing proteins have been proposed to transduce their signals through conformational changes coupled to ligand binding or dissociation, the details of these conformational changes are still being determined.^1−8^ Photoacoustic calorimetry (PAC) has been used to study conformational changes in the nanosecond to microsecond time scale for a variety of heme proteins after ligand photodissociation.^45−48^ In this report, PAC studies are used to generate the thermodynamic profiles for the CO ligand escaping from the heme of the following proteins: the truncated heme domain *Sm*FixLH_128–264_, *Sm*FixL* wild-type with the heme, coiled-coil linker, and histidine kinase domains, but with the N-terminal membrane anchor removed for solubility, and four different *Sm*FixL*variants R200A(Alanine), R200Q(Glutamine), R200E(Glutamate), and R200H(Histidine). These *Sm*FixL* variant proteins, each prepared in pH 7.8 Tris buffer, are presented in order to have a range of polarities and H-bond abilities to compare to those of wild-type SmFixL*. Another *Sm*FixL* variant, I209M(Methionine), was also studied as Mukai et al. have demonstrated that the conformational changes associated with Ile209 and Ile210 are involved in the kinase activity of *Sm*FixL.^49^ The current paper describes the first thermodynamic study of a full-length FixL protein with the heme-PAS, coiled–coil linker, and kinase domains and the first photothermal study of a full-length heme-PAS protein.

## Materials and Methods

### Site-Directed Mutagenesis of *Sm*FixL*

Wild-type *Sm*FixL* and *Sm*FixL* variant proteins were prepared as previously described.^44^

### Protein Expression and Purification of Truncated Heme Domains, *Sm*FixLH_128–264_ and *Sm*FixL*

The truncated heme domain *Sm*FixLH_128–264_ was expressed, purified, and characterized as previously described.^50^ Wild-type and variant *Sm*FixL* with the N-terminal heme-PAS, the coiled-coil linker, and the kinase domain but without the N-terminal membrane linker (AA’s 1–126) was expressed in *Escherichia coli* and purified as previously described.^44^ Protein samples were exchanged into the appropriate buffer, 20 mM Tris-HCl, 0.1 M NaCl, pH 7.8, using either a 3 or 50 mL Millipore Stirred Ultrafiltration cell under compressed nitrogen and stored in aliquots at −80 °C.

### Protein Characterization

Wild-type and variant *Sm*FixL* proteins were separated on 12 or 15% PAGE Gold Precast Gels from Lonza to assess purity and visualized with Coomassie Brilliant Blue (Biorad) staining using Colorburst (Sigma-Aldrich) electrophoresis markers. The heme content of each batch of purified protein was assayed by the standard pyridine hemochromagen assay using the extinction coefficient of the reduced pyridine complex (34.4 mM^–1^cm^–1^) at 557 nm.^51^ SmFixLH_128–264_ and *Sm*FixL* proteins were also characterized by MALDI-TOF mass spectrometry to confirm that the proteins were intact and pure.^44,50^

### Sample Preparation for PAC Measurements

Samples for PAC were prepared by diluting *Sm*FixL* and *Sm*FixLH_128–264_ proteins into a buffer containing 20 mM Tris (pH 8.0). The deoxy form of the protein was formed by placing the oxy form of each SmFixL protein in a quartz optical cuvette that was then sealed with a septum cap and purged with Argon gas. A fresh dithionite solution was added from a buffered stock solution to obtain a final concentration of ∼6 μM. The CO-bound form was obtained by saturating solutions of the deoxy *Sm*FixL* of SmFixLH_128–264_ with CO, resulting in a final solution CO concentration of 1 mM (1 atm pressure). The protein concentration for PAC samples was ∼5 μM and that for transient absorption was ∼9 μM.

### PAC Methods

PAC measurements were performed by placing a 1 cm × 1 cm quartz cuvette containing 1.0 mL of a sample in a temperature-controlled sample holder (Quantum Northwest) housing a Panametrics V103 transducer. Contact between the cuvette and the detector was facilitated by a thin layer of vacuum grease. Photodissociation of CO was achieved with a 532 nm laser pulse (Continuum Minilite I frequency double Q-switched *Nd:YAG laser*, 6 ns *pulse, <* 80 μJ). The acoustic signal was amplified with an ultrasonic preamp (Panametrics) and recorded using an NI 5102 oscilloscope (15 MHz) controlled by VirtualBench software (National Instrument). The PAC data were analyzed using the multiple temperature method, in which sample and calorimetric reference acoustic traces are obtained as a function of temperature. The ratio of the amplitudes of the sample and reference acoustic signals (φ) was then plotted versus *C*_p_ρ/β according to
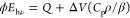
1where ϕ is the ratio of the acoustic amplitudes for the sample and reference (i.e., ϕ = {*A*_S_/*A*_R_}), *Q* is the heat released to the solvent, β is the coefficient of thermal expansion of the solvent (K^–1^), *C*_p_ is the heat capacity (cal g^–1^ K^–1^), ρ is the density (g mL^–1^), and Δ*V* represents conformational/electrostriction contributions to the solution volume change. A plot of ϕ*E*_hν_ versus *C*_p_ρ/β (varied by changing the solution temperature) gives a straight line with a slope equal to Δ*V* and an intercept equal to the released heat (*Q*). Subtracting *Q* from *E*_hν_ and dividing by the quantum yield gives Δ*H* (i.e., Δ*H* = {*E*_hν_ – *Q*}/Φ) for processes occurring faster than the time resolution of the instrument (<20 ns). The *Q*/Φ values for subsequent kinetic processes represent −Δ*H* for that step (i.e., heat released).

For kinetic events occurring slower than the response time of the piezoelectric crystal (between ∼20 ns and ∼20 μs), the resulting sample acoustic wave is shifted in frequency relative to the reference waveform. In order to extract the relevant *Q*_i_, Δ*V*, and rate constants (*k_i_*) corresponding to the observed kinetic processes, the sample acoustic wave, *E*(*t*)_obs_, is treated as a convolution of an instrument response function, *T*(*t*), and time-dependent heat generating function, *H*(*t*), according to
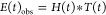
2where
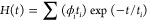
3In practice, the instrument response function *T*(*t*) is taken to be the calorimetric reference waveform. The amplitudes, ϕ*_i_*, and lifetimes, *t_i_*, of the resolvable kinetic processes were extracted by using a simplex parameter estimation algorithm within software developed in our laboratory. Subtracting *Q*_P_ (obtained from the <20 ns phase) from *E*_hν_ and scaling to the reaction quantum yield (Φ) gave the reaction enthalpy (Δ*H*_P_) for the initial (prompt) phase of the reaction, while *Q_i_* = −Δ*H_i_* for each additional step resolved in the deconvolution. The corresponding Δ*V* values for the *Sm*FixL* proteins and the truncated *Sm*FixLH_128–264_ heme domain were obtained from the slopes of the plots described in eq 1 (with the Δ*V* for the prompt phase being the slope/Φ).

The number of kinetic phases is determined through the evaluation of multiparameter fits. Parameter sets for kinetic phases are added until convergence is reached in the χ^2^ values, residuals, and autocorrelation. An example is displayed in the Supporting Information.

### Transient Absorption Methods

Transient absorption (TA) experiments were performed by monitoring the change in intensity of light from a Xe arc lamp (Oriel) emerging from the sample followed by passage through a 1/4 m single monochromator equipped with an Oriel R928 photomultiplier tube. The signal was amplified using a home-built preamplifier (1 MHz bandwidth) followed by a Stanford Instruments SR445A 350 MHz postamplifier. The signal was digitized using a Tektronix TDS7404 4 GHz digital oscilloscope. The sample was excited with the second harmonic of a continuum Leopard I Q-switched mode-locked Nd:YAG laser (<20 ps, 20 mJ/pulse, 20 Hz). Signal traces are the average of 20 laser pulses.

## Results

### Optical Absorption Spectroscopy

**Figure 3:**
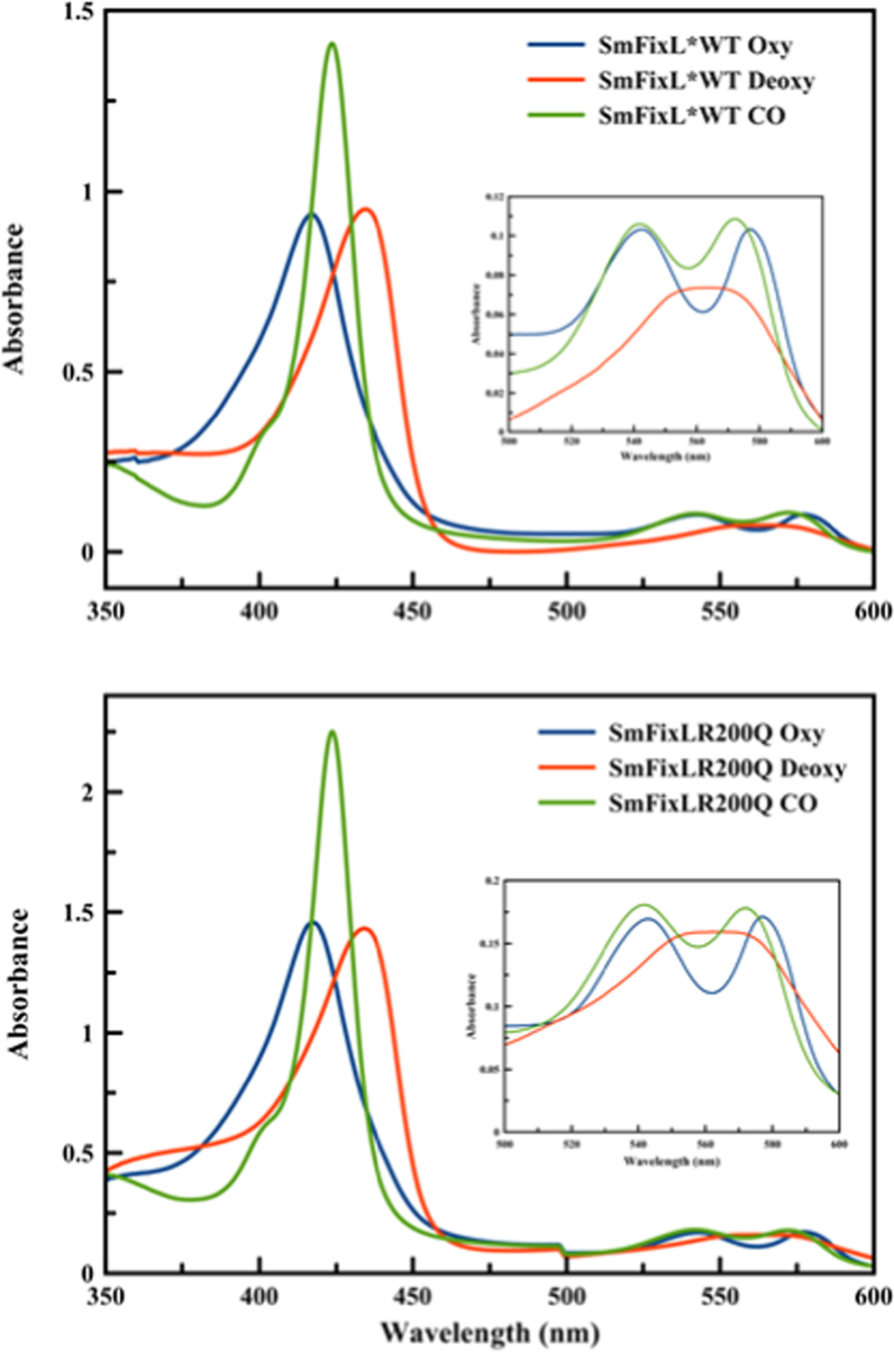
Equilibrium optical absorption spectra of *Sm*FixL*WT (top panel) and *Sm*FixL*R200Q (bottom panel) in the oxy (blue line), reduced deoxy (red line), and CO-bound states (green line). *Sm*FixL*WT and *Sm*FixL*R200Q concentrations: ∼ 5 μM in 20 mM Tris (pH 8).

The UV–vis spectra of *Sm*FixL*WT and R200Q in the as-isolated, the reduced, the deoxy Fe(II)state, and the CO-bound states are shown in Figure 3.

**Figure 4:**
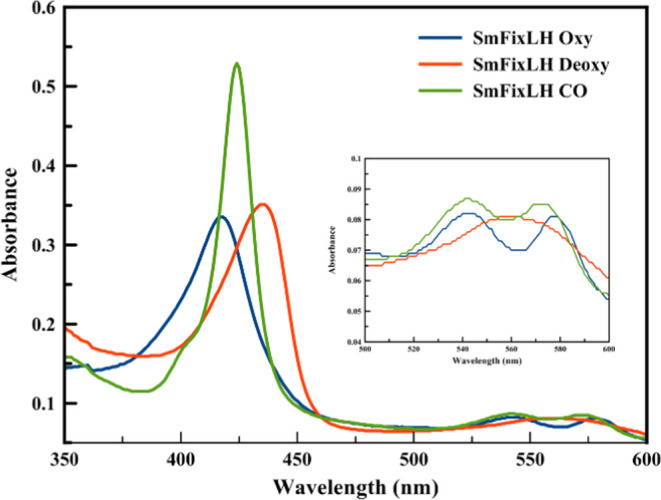
Equilibrium optical absorption spectra of *Sm*FixLH_128–264_ as-isolated (blue line), reduced, deoxy *Sm*FixLH_128–264_ (red line), and reduced CO-bound SmFixLH_128–264_ (green line). SmFixLH_128–264_ concentration: ∼5 μM in 20 mM Tris (pH 8).

The Soret and Q-band wavelengths for the wild-type *Sm*FixL*, the R200 variants, the I209M variants, and the truncated heme domain, *Sm*FixLH_128–264_, are summarized in Table 1. The *Sm*FixL* wild-type and variant proteins (R200A, R200Q, R200E, and R200H) have similar optical spectra, regardless of the change in residue. The optical spectra of the as-isolated *Sm*FixL*WT and R200 variant proteins have Soret bands at ∼418 nm and Q-band at ∼543 and 577 nm indicative of the oxy state. In addition, the as-isolated form of the R200 variants (R200Q/H/A/E) has shoulders at ∼395 nm characteristic of the oxidized, Fe(III) state, which is shown in Figure 3, for R200Q. These R200 variants were previously shown to autoxidize in air during aerobic purification.^44^ The *Sm*FixL*WT and the R200 variant proteins can be reduced to the reduced, Fe(II) state with sodium dithionite, resulting in Soret bands blue-shifted to ∼434 nm with a broad Q-band at ∼566 nm. The binding of CO to the reduced, deoxy Fe(II)*Sm*FixL*WT and R200 variant enzymes results in Soret bands at ∼424 nm and Q bands at ∼542 and ∼572 nm (Figure 3). The fact that the optical absorption spectra of the various forms of *Sm*FixL*WT and R200 variants (R200A, R200Q, R200E, and R200H) are independent of the nature of the variations associated with a range of polarities and H-bond abilities indicate that the salt bridge between the heme-6-propionate and R200 within the distal pocket near the heme group does not have a significant impact on the electronic structure of the heme group. In addition, the truncated heme domain, *Sm*FixLH_128–264_, does not significantly alter the electronic properties of the optical spectra (Figure 4 and Table 1).

**Table 1:** Summary of the Soret and Q Band Wavelength Peaks for Wild-Type *Sm*FixL*WT, R200A, R200Q, R200E, R200H, and I209M Mutants and the Truncated Heme Domain *Sm*FixLH_128–264_

	**as-isolated**			**reduced Form**		**co-bound form**		
**protein**	**soret (nm)**	**α-band (nm)**	**β-band (nm)**	**soret (nm)**	**Q-band (nm)**	**soret (nm)**	**α-band (nm)**	**β-band (nm)**
a*Sm*FixL*WT	418	543	577	434	566	424	542	572
aR200A	418	544	577	434	566	424	542	572
aR200Q	417	543	577	434	565	424	542	572
aR200E	418	543	577	434	565	424	542	572
aR200H	418	544	577	434.4	566	424	542	572
I209M	434	566		434.4	566	424	542	573
*Sm*FixLH_128–264_	418	542	576	577	566	423.5	543	572

a Reynolds et al.^44^

Interestingly, while the *Sm*FixL*I209M variant has similar visible spectra in the reduced, Fe(II) and CO-bound states as *Sm*FixL*WT, the as-isolated *Sm*FixL*I209M variant has a Soret band at ∼434 nm and a broad Q-band centered at ∼557 nm that is indicative of the reduced, Fe(II)state (Supporting Information Figure S1 and Table 1). Thus, as-isolated *Sm*FixL*I209M does not appear to form the same O_2_ complex as *Sm*FixL*WT. It is possible that there is a small amount of the oxy state of SmFixL*I209M that is not readily observable by UV–vis spectrosocopy or that some reduced, Fe(II)*Sm*FixL*I209M forms an oxy complex that is rapidly autooxidized to the Fe(III) state, since we see a small shoulder at ∼395 nm that is indicative of oxidized, Fe(III)*Sm*FixL*. The intense 418 nm Soret peak that is characteristic of the oxy state is not observed, while the CO-bound state of *Sm*FixL*I209M is similar to that of *Sm*FixL*WT(CO) with a peak at 424 nm (Table 1). It appears that while the I209M variant readily binds CO, the change to a larger, more polarizable methionine sulfur group significantly lowers the affinity of reduced Fe(II)*Sm*FixL*I209M for O_2_. This suggests that the conserved isoleucine 209 residue in the distal pocket of *Sm*FixL* plays an important role in O_2_ binding to the heme iron. We are in the process of further characterizing this interesting *Sm*FixL*I209M variant protein.

### Transient Absorption

Photolysis of CO from *Sm*FixL*WT(CO) and the five different variants (R200A, R200Q, R200E, R200H, and I209M) probed at 450 nm results in the formation of a five coordinate high-spin heme complex, which decays back to the preflash CO-bound complex (Supporting Information Figure S2) with monophasic relaxation kinetics. Table 2 summarizes the rate constants for CO rebinding to the previously reported truncated heme domains of *Bj*FixLH_140–270_, and *Bj*FixLH_151–256_, and the full-length *Sm*FixL*WT and the five variants R200A, R200Q, R200E, R200H, and I209M. The truncated heme domain *Bj*FixLH_140–270_ had a CO rebinding rate constant equal to 10.2 ± 0.3 s^–1^, while the shorter truncated heme domain, *Bj*FixLH_151–256_, was equal to 17.3 ± 0.1 s^–1^.^41^ Full-length *Sm*FixL*WT with the heme and kinase domain and the five different variants had rate constants between 33 and 41 s^–1^. The results show a faster CO rebinding rate constant for full-length *Sm*FixL*WT and the R200 variants compared to the one observed for the truncated heme domains of *Bj*FixLH_140–270_ and *Bj*FixLH_151–256_. These results indicate that the full-length *Sm*FixL* protein accelerated the rebinding of CO, and they also demonstrate that a change at R200 does not significantly affect the rebinding of CO to the heme relative to *Sm*FixL*WT. The rate constant associated with CO rebinding to *Sm*FixL*I209M is slightly slower than *Sm*FixL*WT, which may be due to the fact that the methionine sulfur group could interact with the CO molecule, compared to the alkyl group in isoleucine, which has a steric repulsion with CO. It is also possible that differences in the hydrogen-bonding networks in the heme pockets of full-length *Sm*FixL* and the truncated heme domains of *Bj*FixLH could affect the CO rebinding rates observed.

**Table 2:** Summary of the Rate Constants Associated with CO Rebinding to *Sm*FixL*WT, R200A, R200Q, R200E, R200H, and I209M and the Truncated Heme Domains *Bj*FixLH_140–270_ and *Bj*FixLH_151–256_^a^

**protein**	***k***_**1**_**(s**^**–1**^**)**	**refs**
*Sm*FixL*WT	37.3 ± 0.1	this work
R200A	38 ± 0.1	this work
R200Q	36.5 ± 0.1	this work
R200E	41.3 ± 0.1	this work
R200H	40.5 ± 0.4	this work
I209M	33.1 ± 0.4	this work
BjFixLH_140–270_	10.2 ± 0.3	(41)
BjFixLH_151–256_	17.3 ± 0.1	(41)

a Data was obtained with 532 nm excitation at 20 °C.

### Photoacoustic Calorimetry

**Figure 5:**
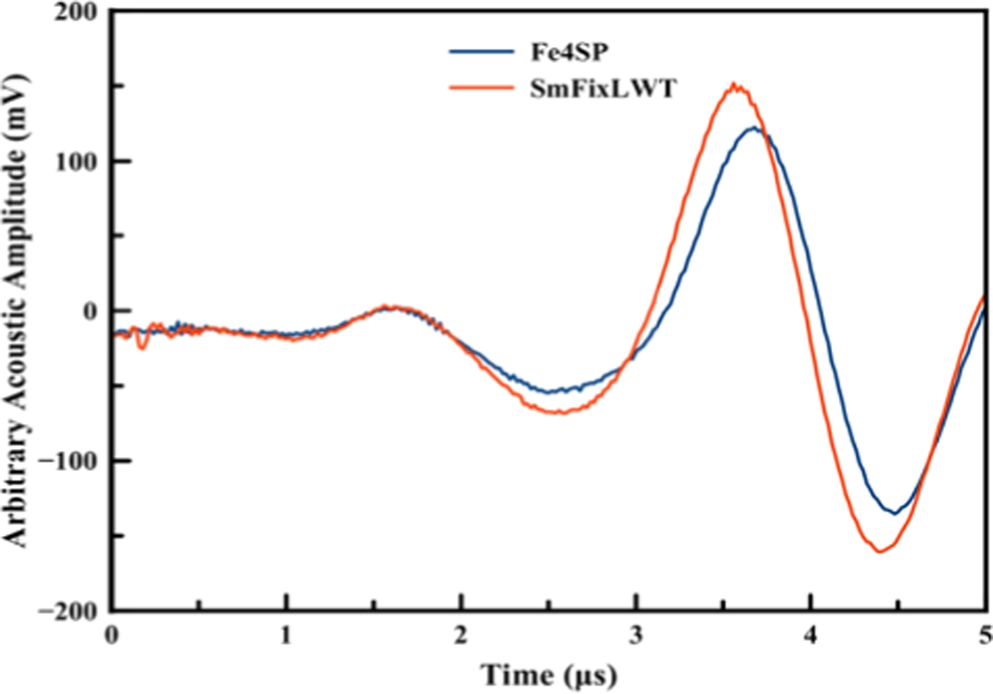
Overlay of the acoustic waves for the photolysis of CO from *Sm*FixL*WT (red solid line) and the reference Fe(III)4SP (blue solid line).

**Figure 6:**
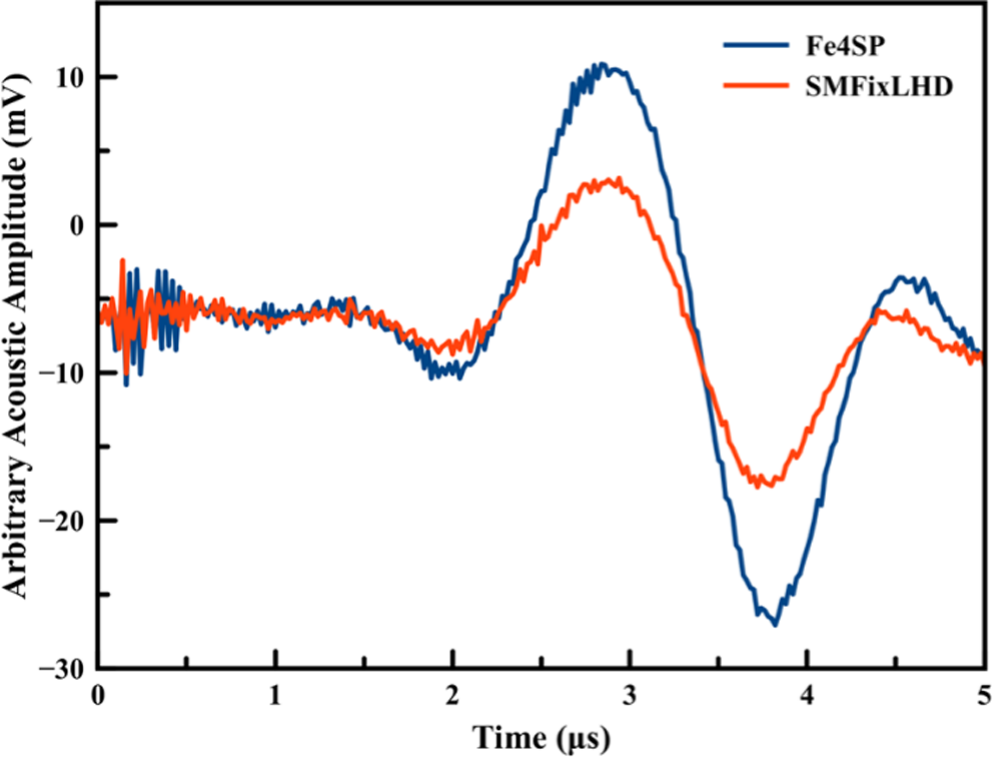
Overlay of the acoustic waves for the photolysis of CO from the truncated heme domain *Sm*FixLH_124–264_ (red solid line) and reference Fe(III)4SP (blue solid line).

Figures 5 and 6 display representative PAC traces for CO bound to *Sm*FixL*WT and *Sm*FixLH_128–264_ and the calorimetric reference compound Fe4SP obtained in 20 mM Tris buffer, pH 8.0.

**Figure 7:**
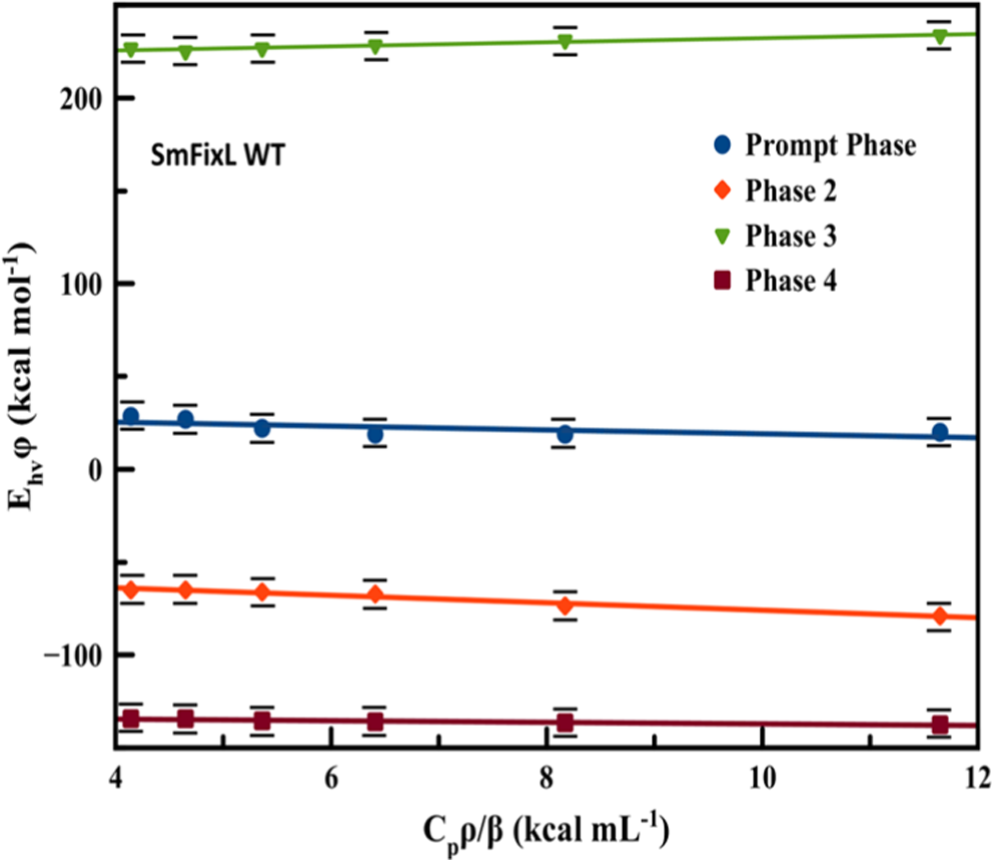
Plot of (S/R)* E*h*ν versus *C*_p_ρ/β for CO photolysis from *Sm*FixL*WT in 20 mM Tris (pH 8) between 10 and 34 °C.

The fact that a frequency shift is observed between the sample and reference acoustic signals indicates that kinetic events occur between <20 ns and ∼20 μs. The deconvolution of the acoustic wave between the sample and the reference results in four kinetic phases with average lifetimes of <20 ns (prompt phase), ∼190 ns, ∼512 ns, and ∼1.5 μs. The reaction volume and enthalpy changes were calculated using an Φ of 0.86 determined by Rodgers et al.^52^ A plot of φE_hν_ versus *C*_p_ρ/β for the four observed intermediates is shown in Figure 7 for full-length *Sm*FixL*WT.

The calculated Δ*H* and Δ*V* values for each intermediate associated with CO photodissociation from *Sm*FixL*WT, the R200 and I209M variants, and the truncated heme domains of *Sm*FixLH and *Bj*FixLH are shown in Table 3. The additional plots of φ*E*_hν_ versus *C*_p_ρ/β for the *Sm*FixL* variants are displayed in the Supporting Information.

**Table 3:** Summary of the Photoacoustic Rresults for *Sm*FixL*WT, R200A, R200Q, R200E, R200H, and I209M^a^

**protein**	τ_1_ (ns)	Δ*V*_1_ (mL mol^–1^)	Δ*H*_1_ (kcal mol^–1^)	τ_2_ (ns)	Δ*V*_2_ (mL mol^–1^)	Δ*H*_2_ (kcal mol^–1^)	τ_3_ (ns)	Δ*V*_3_ (mL mol^–1^)	Δ*H*_3_ (kcal mol^–1^)	τ_4_ (ns)	Δ*V*_4_ (mL mol^–1^)	Δ*H*_4_ (kcal mol^–1^)
*Sm*FixL*WT	<20	10 ± 3	–9 ± 8	190	–18 ± 6	18 ± 16	512	11 ± 6	–35 ± 20	1495	–9 ± 8	31 ± 25
R200A	<20	15 ± 2	–7 ± 6	100	–6 ± 4	63 ± 20	510	28 ± 6	–51 ± 20	1510	–10 ± 3	18 ± 11
R200Q	<20	17 ± 2	–26 ± 13	92	–19 ± 1	62 ± 8	285	8 ± 0.9	–43 ± 5	1252	–7.6 ± 0.6	9 ± 3
R200E	<20	21 ± 3	–10 ± 5	120	–21 ± 2	31 ± 14	429	10 ± 1	–47 ± 6	1575	–5 ± 2	43 ± 10
R200H	<20	4.7 ± 0.7	–39 ± 5	95	–7 ± 1.1	74 ± 7	365	3.9 ± 0.7	–49 ± 5	1608	–4.3 ± 0.7	75 ± 5
I209M	<20	3 ± 0.4	–54 ± 3	115	–3.7 ± 1	84 ± 6	497	5.7 ± 2	–30 ± 13	1493	–7.8 ± 0.8	14 ± 5
*Sm*FixLH_128–264_	<50	21 ± 6	8.8 ± 0.9									
b*Bj*FixLH_140–270_	<50	–1.4 ± 0.8	14 ± 3	150	5.3 ± 0.7	6.0 ± 3.5						
b*Bj*FixLH_151–256_	<50	25 ± 4.0	4.9 ± 0.4									

a Lifetime values are reported for the 20°C deconvolutions

b Miksovska et al.^41^

## Discussion

Previous studies using time-resolved crystallography demonstrated that within 1 μs after CO photolysis, the truncated heme domain, *Bj*FixLH_141–270_, has relaxed to a conformation of the protein, which is identical to the deoxy Fe(II) state.^30^ These studies also demonstrated that the transmission of the signal after photodissociation of CO is not restricted to a single region surrounding the heme but to an ensemble of regions, including the FG loop and the β-sheet distal of the heme involving the movement of Arg206. Photodissociation of CO from *Bj*FixLH also results in the doming and displacement of the heme, and the collapse of the hydrophobic residues Leu 236, Ile 215, and Ile 238 in the distal pocket in order to fill the space left after CO leaves the heme pocket. Furthermore, the displacement of the heme-6-propionate group and the FG loop residues Pro212, His213, and Ile216 is notable, as are the conformational changes in the proximal histidine and the Fα-helix backbone atoms of the H and I β-strands with Leu 236 and Val 253 on the surface of the protein. Structural similarities between *Bj*FixL and *Sm*FixL suggest that these conformational changes may also occur after the photo cleavage of CO from the heme of *Sm*FixL.

The overall Δ*V* and Δ*H* values for each phase are related to (1) a change in overall charge distribution on the protein (i.e., change in net protein dipole leading to solvent reorganization), (2) formation of one or more salt-bridge interactions (the release of electrostricted water molecules upon salt-bridge formation results in volume increases), and/or (3) an increase in the solvent accessible van der Waals volume of the protein immediately upon photolysis.

### Truncated *Sm*FixLH_128–264_ Heme Domain

Photolysis of CO from the truncated *Sm*FixLH_128–264_ heme domain results in a monophasic relaxation after the photodissociation of CO with a Δ*H* of ∼9 kcal mol^–1^ and Δ*V* of ∼22 mL mol^–1^. CO photolysis of the heme domain of *Sm*FixLH_128–264_ produces thermodynamics distinct from those from the heme domain of FixL from *B. japonicum* (*Bj*FixLH_140–270_), which display a biphasic relaxation after the photodissociation of CO.^41^ For *Bj*FixLH_140–270_, the fast phase (Δ*H* of −1.4 kcal mol^–1^ and Δ*V* of 14 mL mol^–1^) was associated with a reorganization of the solvent, following a perturbation to the salt bridge between Glu182 and Arg227.^41^ The corresponding slow phase with a lifetime τ ∼ 150 ns and a Δ*H* of ∼6 kcal mol^–1^ and Δ*V* of ∼5 mL mol^–1^ was associated with the escape of the CO molecule to the solvent.^41^ In contrast, the thermodynamics of CO release from *Sm*FixLH_128–264_ is more similar to photolysis from the more truncated heme domain *Bj*FixLH_151–256_ with Δ*H* ∼ 5 kcal mol^–1^ and Δ*V* ∼ 25 mL mol^–1^ (Table 3). The truncated *Bj*FixLH_151–256_ heme domain has an additional 11 amino acid residues deleted from the N-terminus and 14 amino acid residues deleted from the C-terminus. Miksovska et al. concluded that the truncation of these additional N-terminal and C-terminal amino acids from the heme-PAS domain of *Bj*FixLH_151–256_ induces changes associated with the protein surface that accelerate the release of the ligand from the protein and/or change the salt-bridge interactions.^41^ The results obtained for the variation of volume are similar between *Bj*FixLH_151–256_ and *Sm*FixLH_128–264_, but the variation in enthalpy is nearly 2-fold lower, which may arise from the fact that the salt bridge does not involve the same amino acids for the different proteins.

### Full-length *Sm*FixL*

**Figure 8:**
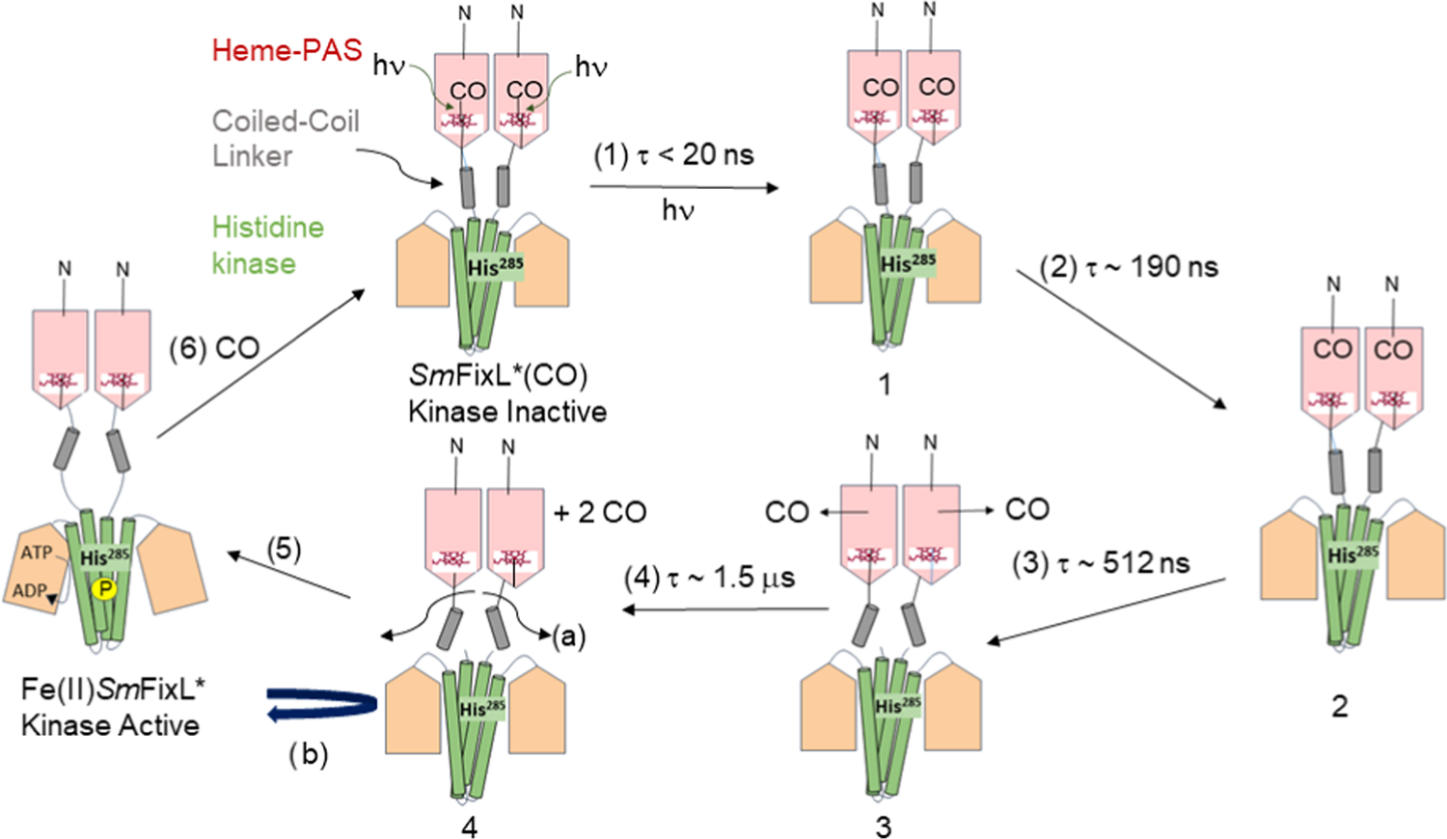
Proposed mechanism for the escape of CO from the heme of *Sm*FixL*(CO) after photodisssociation. CO is photolyzed from the heme of *Sm*FixL*(CO) in <20 ns in step (1) to form transient intermediate 1, which is the prompt phase. In step (2), CO migration occurs within the heme domain of *Sm*FixL* with the conserved hydrophobic residues of the distal heme pocket (Ile210, Val232, Ile209, and Leu230) potentially forming an initial transient CO binding site, Intermediate 2, after ∼190 ns. In step (3), CO would then migrate from the initial heme domain transient binding site to the bulk solvent to form intermediate 3 at ∼512 ns. The initial conformational changes that are propagated from the heme domain to the coiled-coil linker domain in steps 1 to 3 result in the formation of intermediate 4 in step (4) at ∼1.5 μs, which could result in a (b) right-handed supercoiled rotation of the histidine kinase domain of *Sm*FixL*, similar to the one observed at ∼2 μs for the chimeric YF1 protein that contains the histidine kinase domains of *Bj*FixL.^64^ In step 5, the right-handed supercoiled rotation could cause a rearrangement of the histidine kinase domains of *Sm*FixL*, as observed in the chimeric YF1 protein after 250 ms, to form kinase active, deoxy Fe(II)*Sm*FixL*.^64^ Kinase active, deoxy Fe(II)*Sm*FixL* can phosphorylate *Sm*FixJ, the response regulator, which triggers the expression of FixK2, the transcriptional activator of the nitrogen fixation genes.^13−17^ In step 6, additional CO can be added to reform kinase inactive *Sm*FixL*(CO) through additional conformational changes.

Photolysis of CO from the heme of *Sm*FixL*WT with the heme, coiled-coil linker and kinase domains, results in four intermediates between <20 ns and ∼2.0 μs with lifetimes of <20 ns (prompt phase), ∼ 190 ns, ∼ 512 ns, and ∼1.5 μs. With regard to the prompt phase (<∼20 ns), the volume and enthalpies are distinct from those observed for the truncated heme domain, *Sm*FixLH_128–264_, with a Δ*H* of ∼ - 9 kcal mol^–1^ for the full-length *Sm*FixL*WT protein versus Δ*H* of ∼9 kcal mol^–1^ for the *Sm*FixLH_128–264_ heme domain and Δ*V* of ∼10 mL mol^–1^ for the full-length *Sm*FixL*WT protein versus Δ*V* of ∼21 mL mol^–1^ for *Sm*FixLH_128–264_, suggesting that the initial response of the heme domain to ligand release is linked to conformational changes involving both the coiled-coil linker and kinase domains. The initial conformational changes associated with the heme domain after CO photolysis are followed by three additional intermediates with lifetimes of ∼190, 512, and 1.5 μs with Δ*H* and Δ*V* values summarized in Table 3. Time-resolved photothermal methods have also revealed two phases of similar lifetimes subsequent to the photolysis of CO from horse heart myoglobin (HH Mb) with lifetimes of ∼180 ns and ∼400 ns.^53−60^ The time-resolved transient grating studies assigned the ∼180 ns lifetime to the migration of the photocleaved CO to a hydrophobic Xe binding pocket near the heme iron made up of distal residues Leu29, Ile107, and Val68.^60^ The corresponding ∼400 ns phase arises from CO migration from the Xe binding pocket to the exterior of the protein with additional water entry into the heme pocket.^53−60^ It is possible that CO migration occurs within the heme domain of *Sm*FixL* with the conserved hydrophobic residues of the distal pocket (Ile210, Val232, Ile209, and Leu230), forming an initial transient CO binding site (Figure 8).

The initial migration of CO from the heme to the hydrophobic pocket would then be followed by the migration of CO through this cavity to the bulk solvent. The endothermic nature of this ∼190 ns CO migration phase indicates that this is largely entropically driven, while the corresponding CO release to the bulk solvent at ∼512 ns is an enthalpy-driven event that likely involves protein reorganization. The relatively small volume change for CO release to the solvent (∼+35 mL mol^–1^) is likely due to the corresponding entry of a water molecule to the pocket (∼ – 18 mL mol^–1^). The fact that these phases which are assigned here to heme domain CO release events only occur in the full-length *Sm*FixL* protein, and not with the truncated heme domain *Sm*FixLH_128–264_, suggests that the coiled-coil linker region connecting the heme domain to the kinase domain may be essential to regulating ligand binding and release and couple these events to histidine kinase domain conformational changes. Recent structural and site-directed mutagenesis studies of the full-length FixL_2_-FixJ_2_ dimer from *B. japonicum* showed that the coiled-coil linker domain is important for signal transduction between the heme-PAS sensor and the histidine kinase domains.^32^ Changing 11 of 12 different amino acids in the coiled-coil linker of full-length *Bj*FixL resulted in significantly impaired kinase activity.^32^ Conformational changes in the coiled-coil linker domain of the heterodimeric, eukaryotic NO-sensing (H-NOX) heme protein soluble guanylate cyclase (sGC), which controls blood pressure, were also recently shown to regulate enzymatic activity.^61−63^ The straightening of the coiled-coil linker domain of sGC upon activation with NO or an inserted leucine zipper was sufficient to explain the elevated guanylate cyclase activity.^61−63^

The slowest phase of CO photodissociation in *Sm*FixL* occurs on a time scale similar to that reported for O_2_ photodissociation obtained from time-resolved UV resonance Raman spectroscopy (1.5 μs from PAC versus 3 μs from UV resonance Raman).^36^ Interestingly, the UV resonance Raman study does not show such a phase for CO photodissociation. It is possible that signal transmission from the heme domain may involve different pathways depending on the ligand type. The UV resonance Raman probed the Tyr8a band of Tyr201 that forms a hydrogen bond with Glu234. Upon photodissociation of O_2_, this H-bond is disrupted, which contributes, in part, to the signal migration.^36^ The 1.5 μs slow phase of *Sm*FixL* also correlates with recent time-resolved solution X-ray scattering experiments with the chimeric YF1 protein that contains a LOV photosensor PAS domain, a coiled-coil linker, and the histidine kinase domain of FixL from *B. japonicum* that is kinase active in the dark.^64^ They found that the coiled-coil linker and histidine kinase domain of dimeric YF1 undergo a left-handed supercoiled rotation that is complete 2 μs after blue light excitation, which triggers a rearrangement of the histidine kinase domains after 250 ms and results in kinase inactivation.^64^ In contrast, a H22P variant of YF1 is kinase inactive in the dark state but becomes kinase active after exposure to blue light with inverted sign polarity, suggesting that the H22P YF1 variant instead undergoes a right-handed supercoiled rotation which activates the kinase domains.^64,65^ If *Sm*FixL behaves in a manner similar to that of the H22P YF1 variant protein, we would predict that the loss of CO from the heme of *Sm*FixL would result in a right-handed supercoiled rotation activating the histidine kinase domains. Our PAC experiments suggest that the initial conformational changes we observe upon CO photolysis from the heme domain of *Sm*FixL* are propagated to the histidine kinase domain through the coiled-coil linker domain to form a similar ∼1.5 μs intermediate. More studies are needed to determine whether FixL undergoes the same supercoiled rotation and histidine kinase rearrangement as the chimeric YF1 protein and whether this regulates the histidine kinase activity of FixL.

### *Sm*FixL*R200 and I209 Variants

The corresponding thermodynamics associated with *Sm*FixL*WT and the R200A, R200Q, R200E, R200H, and I209M variants all display four kinetic phases with lifetimes similar to that observed for the *Sm*FixL*WT protein (Table 3). As can be seen from Table 3, all full-length *Sm*FixL* variant proteins display exothermic prompt phases (τ < 20 ns), followed by endothermic ligand migration phases (τ_avg_ ∼ 104 ns), exothermic ligand release phases (τ_avg_ ∼ 417 ns), and endothermic conformational change phases (τ_avg_ ∼ 1.49 μs). However, the Δ*H* values vary significantly among the protein variants. The initial phases for the R200A and R200E have similar Δ*H* values to WT, while the R200Q, R200H and I209M variants all have significantly lower values. This suggests minimal perturbation to the hydrogen-bonding network surrounding the heme-6-propionate network for the R200A and R200E variants, but some disruption occurs for the R200Q and R200H variants. The thermodynamics associated with the variants further demonstrate that the R200 and heme-6-propionate H-bond network may also play a role in stabilizing the transient CO binding to the distal hydrophobic pocket with the most pronounced perturbation from the I209M variant, as might be expected as this residue contributes to the putative transient CO binding pocket. Interestingly, the R200A, R200Q, and I209M variants have much lower Δ*H* values, indicating that a more entropically driven process drives the conformational response, while the opposite is true for the R200E and R200H variants.

The corresponding volume changes are also widely variable between the variants and the WT protein. Volume changes largely arise from protein–solvent interactions and are most influenced by changes in the overall surface charge. The magnitude of molar volume changes is dependent largely on the extent of changes in surface charge exposure. In the case of the *Sm*FixL* variants, the R200A, R200Q, and R200E variants give rise to increase in Δ*V* upon initial CO photolysis, which is likely due to surface charge reduction (i.e., reducing solvent–residue electrostriction) and is consistent with the modest changes in the Δ*H* values (i.e., greater electrostriction results in greater charge stability and more negative Δ*H* values). In contrast, the R200H and I209M variants have lower Δ*V* values, indicating more exposed charge and more negative Δ*H* values. The Δ*V* associated with migration of CO within the protein interior for the R200Q and R200A variants is similar to that of WT, while the remaining variants have less negative Δ*V* values consistent with overall charge reduction. The CO release process gives rise to Δ*V* values similar for all variants except for R200A. Interestingly, the longer time scale conformational change gives Δ*V* values similar to WT (R200A and I209M) and values more positive than WT (R200E, R200H, and R200Q). These trends also track well with the observed Δ*H* values.

Although the time-resolved thermodynamics presented here lack the atomic level detail required for detailed mechanistic analysis, they do provide important insights into the mechanism of FixL signaling. The data presented here for full-length *Sm*FixLWT* and five variant proteins are the first to demonstrate multiphasic response to ligand release from the heme domain with four intermediates observed from <20 ns to ∼1.5 μs. These studies also provide important thermodynamic boundaries for future studies of the signal transduction mechanism of FixL and other heme-PAS proteins.

## Conclusions

In summary, PAC studies reveal a quadriphasic relaxation for full-length *Sm*FixLWT* and the five different variants (*Sm*FixL*R200A, *Sm*FixL*R200Q, *Sm*FixL*R200E, *Sm*FixL*R200H, and *Sm*FixL*I209M) with four intermediates from <20 ns to ∼1.5 μs associated with the photodissociation of CO from the heme. The thermodynamic profiles of the R200 and I209M variant proteins confirm that these conserved residues are important in the transmission of the signal and that changing either residue results in altered intermediates, which may cause a different transmitted signal. The results for the truncated heme domain *Sm*FixLH_128–264_ instead show a monophasic relaxation at <50 ns associated with a fast disruption of the salt bridge and release of CO to the solvent, suggesting that the full-length protein is necessary to observe the conformational changes that are transmitted from the heme domain through the coiled-coil linker to the kinase domain. These are the first studies to observe multiple conformational changes in a full-length FixL protein and are a model for the ubiquitous heme-PAS and histidine kinase families of signaling proteins.

## Supporting Information Available

Additional figures including the electronic spectrum of as-isolated *Sm*FixL*I209M, the transient absorption data for CO recombination, the photoacoustic waves of *Sm*FixL* variant proteins and the plots of (S/R)* Ehν versus *C*_p_ρ/β for CO pho*tolysis from SmFixL* variants and a fit of the SmFixLWT** PAC data (PDF)

## Accession Codes

FixL from *Sinorhizobium meliloti* (formerly *Rhizobium melioti*), P10955.

## Abbreviations

Bj: *Bradyrhizobium japonicum*
BjFixLH: the truncated heme domain-only form of BjFixL without the kinase domain
CC: coiled-coil linker
Fe4SP: Fe^3+^ tetrakis(4-sulfonatophenyl)porphine
GAF: cGMP-specific and stimulated phosphodiesterases, adenylate cyclases, and *E. coli* formate hydrogen lyase transcriptional activator
GCS: Globin coupled sensor
HD: Heme Domain
HH: horse heart
HK: histidine kinase
H-NOX: heme-NO and oxygen-binding domain
LOV: light-oxygen-voltage sensing domain
MALDI-TOF: matrix-assisted laser desorption ionization-time-of-flight
PAC: photoacoustic calorimetry
PAS domain: sensory domain with an α–β fold named after the period circadian (Per), aryl hydrocarbon receptor nuclear translocator (Arnt), and single-minded (Sim) eukaryotic proteins
Sm: *Sinorhizobium meliloti*
SmFixLH_128–264_: the truncated heme domain of SmFixL
SmFixLWT*: FixL from *S. meliloti* contains the N-terminal heme-PAS domain, the coiled-coil linker domain the kinase domain but is missing the N-terminal transmembrane helices, residues 1–126; instead, there is an N-terminal extension: (N-TMITPSLAAGR(127–505)-C)^20^
SAS: solvent accessible surface
WT: wild-type
