# Supplementary material for: Photoacoustic Calorimetry Studies of O2-Sensing FixL and (R200, I209) Variants from Sinorhizobium meliloti Reveal Conformational Changes Coupled to Ligand Photodissociation from the Heme-PAS Domain
Source: Biochemistry. 2023 Dec 21;63(1):116–27. doi: 10.1021/acs.biochem.3c00438 (PMC10765370; doi:10.1021/acs.biochem.3c00438)
Supplement: Supplementary file 1 — bi3c00438_si_001.pdf [file bi3c00438_si_001.pdf]

Supporting Information

Photoacoustic Calorimetry Studies of O_2-Sensing FixL and (R200, I209) Variants from *Sinorhizobium meliloti* Reveal Conformational Changes Coupled to Ligand Photodissociation from the Heme-PAS Domain

Audrey Mokdad[a], EuTchen Ang[b], Michael Desciak[b], Christine Ott[b], Avery Vilbert[b], Olivia Beddow[b], Artiom Butuc[b], Randy W. Larsen[a], and Mark F. Reynolds[b]*

[a]Department of Chemistry, University of South Florida, 4202 East Fowler Avenue SCA 400, Tampa, Florida 33620

[b]Department of Chemistry and Biochemistry, Saint Joseph's University, 5600 City Avenue, Philadelphia, PA 19131

*Corresponding author

*email: mreynold@sju.edu

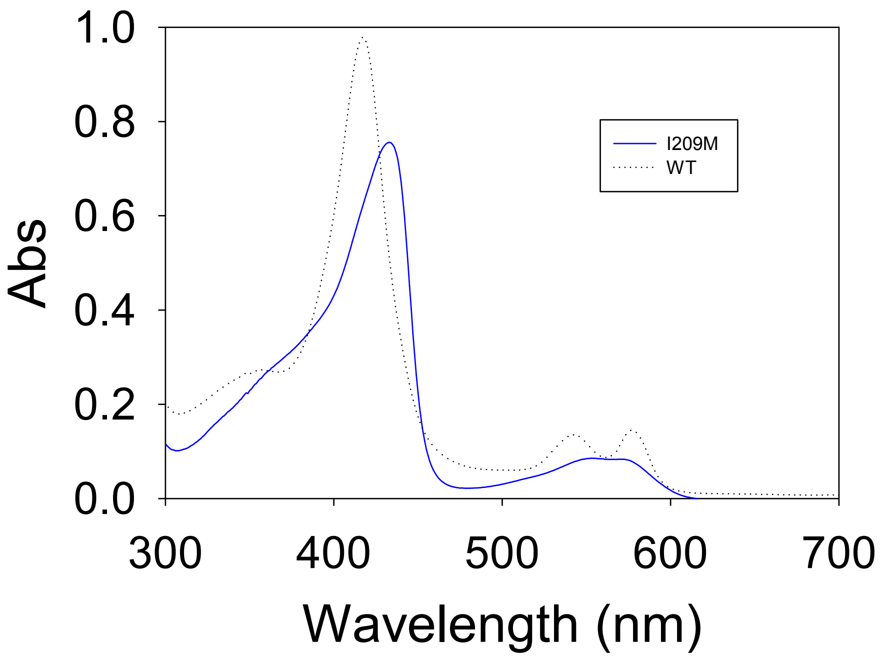

Figure S1. Equilibrium optical absorption spectra of as isolated SmFixL*WT (red dots) and SmFixL*I209M (blue solid).

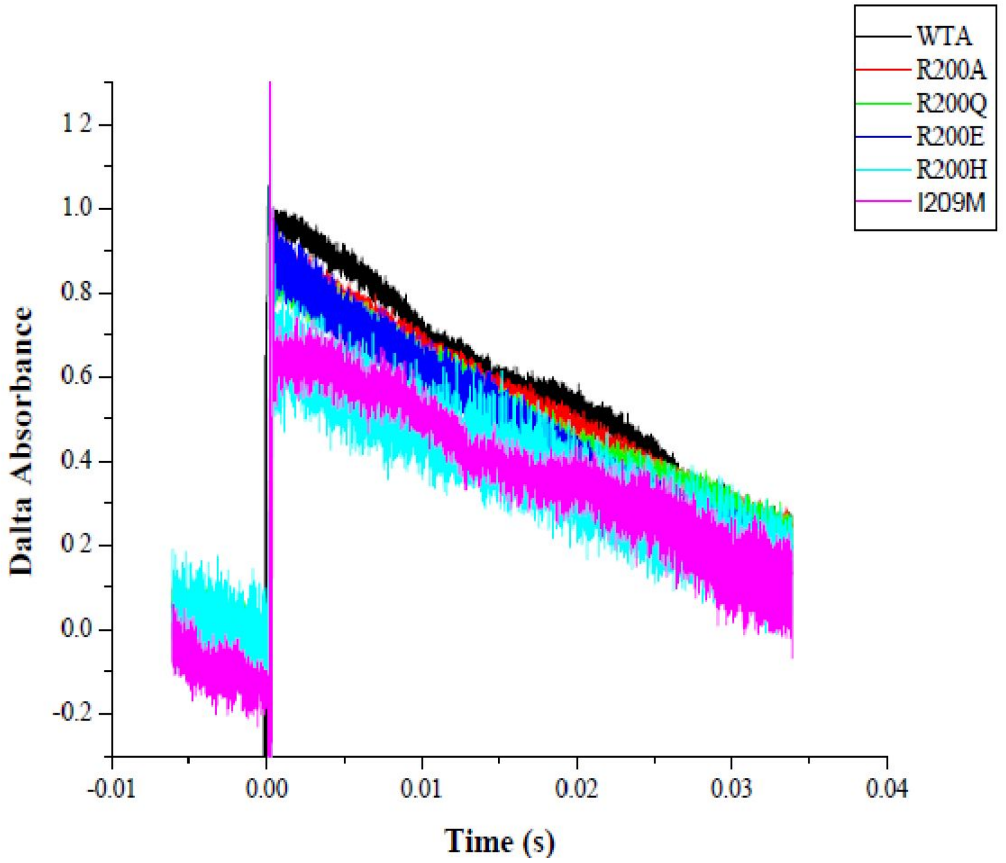

Figure S2. Single wavelength transient absorption data for CO recombination to *Sm*FixL*WT, *Sm*FixL*R200A, *Sm*FixL*R200Q, *Sm*FixLR200*E, *Sm*FixL*R200H and *Sm*FixL*I209M at 25ºC. Excitation wavelength was 532 nm (<20 ps, 20 mJ/pulse, 20 Hz). Sample solution conditions are the same as those reported in Figure 3. Time scale: 40 ms.

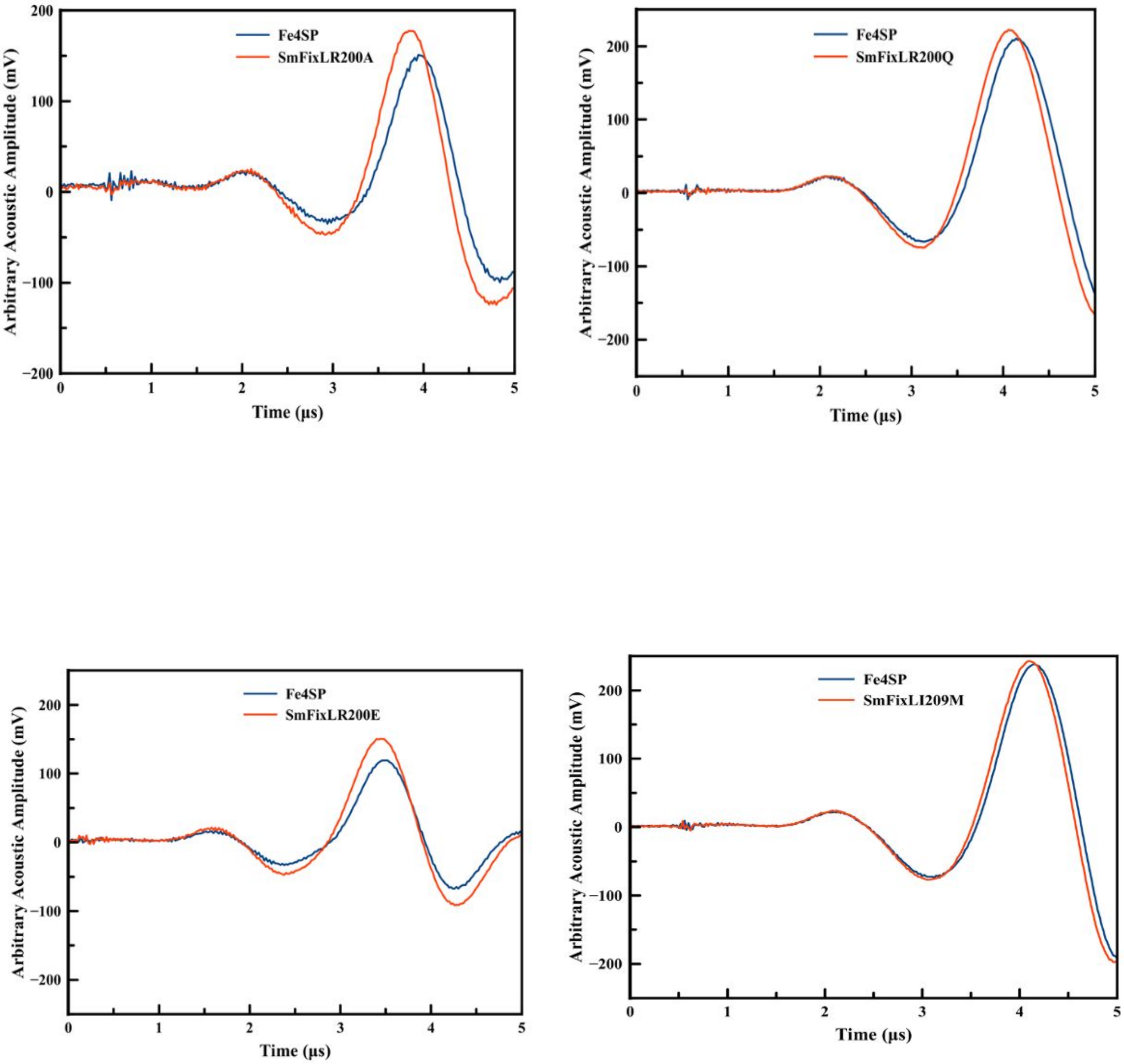

Figure S3. Overlay of the acoustic waves for the photolysis of CO from SmFixL* variants (R200A, R200E, R200Q and I209M), and the reference Fe(III)4SP (blue solid line).

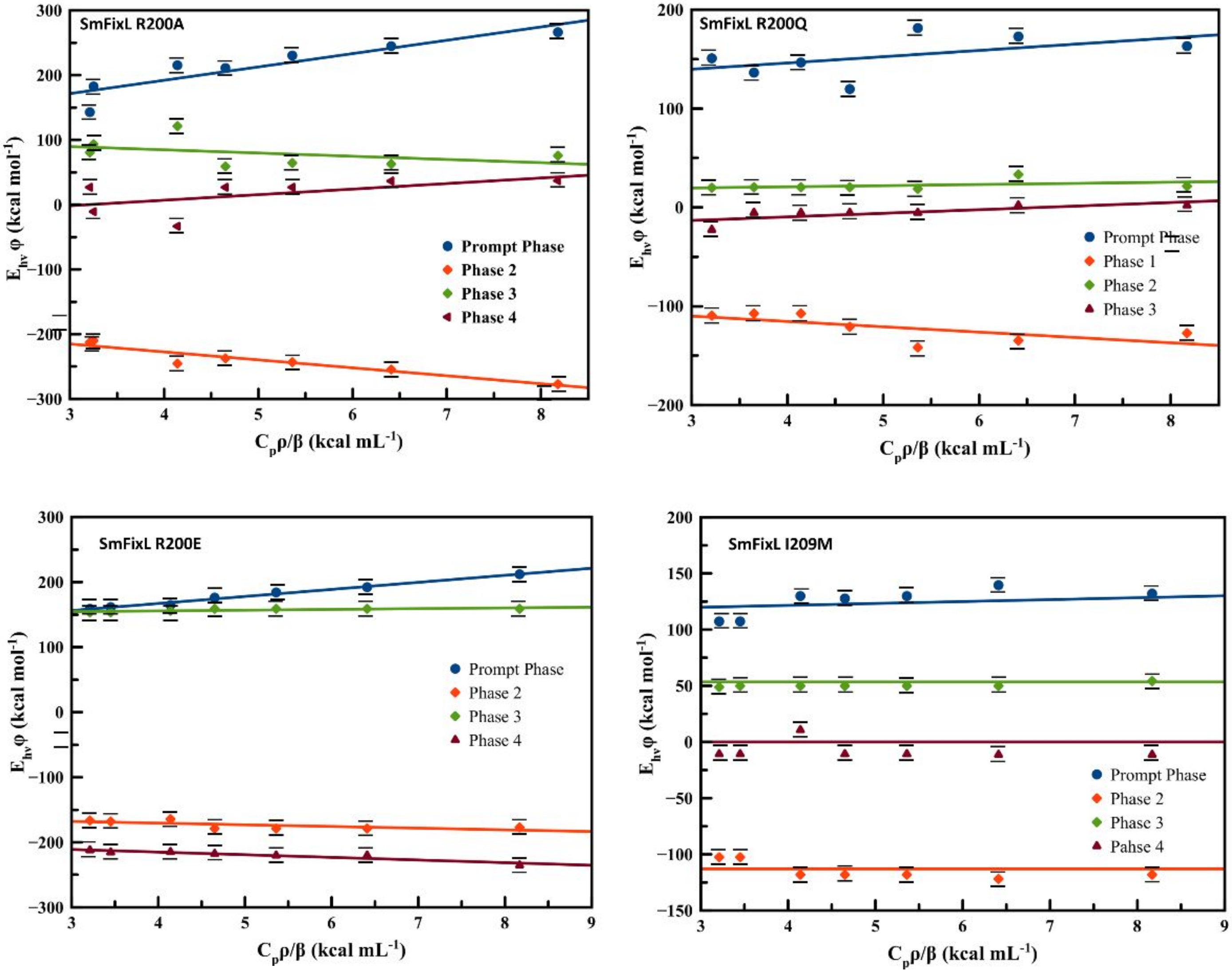

Figure S4. Plot of (S/R)* Ehν versus $C_p\rho/\beta$ for CO photolysis from *Sm*FixL variants in 20 mM Tris (pH 8) between 10 °C and 34 °C.

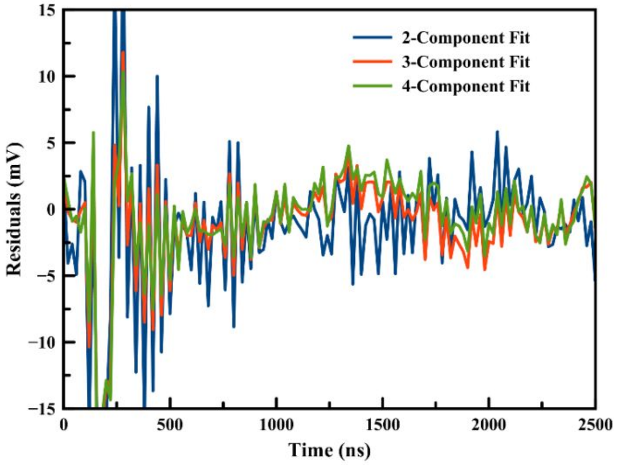

.

Figure S5. Representative examples of the residuals (left) and autocorrelation (right) for 2-, 3- and 4-components fits for SmFixLWT at 34 ˚C. The χ^2 values for each fit are: 2.9 (2-component), 1.57 (3-component) and 1.4 (4-component). No further improvement is observed beyond a 4-component fit.
